# Supplementary material for: MEANtools integrates multi-omics data to identify metabolites and predict biosynthetic pathways
Source: PLoS Biol. 2025 Jul 28;23(7):e3003307. doi: 10.1371/journal.pbio.3003307 (PMC12327601; doi:10.1371/journal.pbio.3003307)
Supplement: S1 Fig — Loose dataset contains reaction rules-enzyme associations from the RetroRules database, cross-referenced with the Rhea and KEGG-orthology database. Medium dataset contains experimentally validated entries together with the ECDomainMiner predictions. The strict dataset contains only experimentally validated entries. The raw data underlying the Venn diagram can be found at https://zenodo.org/records/15697913/files/Combined_small_middle_big_datasets.csv (DOCX) [file pbio.3003307.s001.docx]

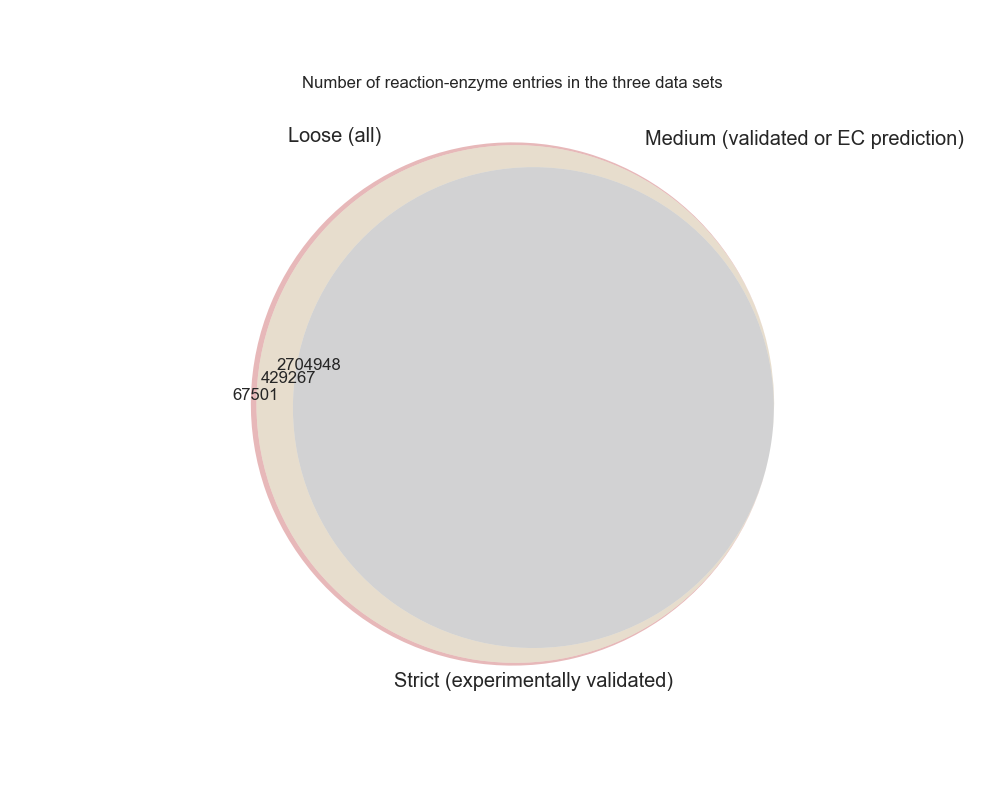


**S1 Fig**: Venn diagram of the content of three datasets, namely loose, medium and strict. *Loose* dataset contains reaction rules-enzyme associations from the RetroRules database, cross-referenced with the Rhea and KEGG-orthology database. *Medium* dataset contains experimentally validated entries together with the ECDomainMiner predictions. The *strict* dataset contains only experimentally validated entries. The raw data underlying the Venn diagram can be found at https://zenodo.org/records/15697913/files/Combined_small_middle_big_datasets.csv
