# Supplementary material for: MEANtools integrates multi-omics data to identify metabolites and predict biosynthetic pathways
Source: PLoS Biol. 2025 Jul 28;23(7):e3003307. doi: 10.1371/journal.pbio.3003307 (PMC12327601; doi:10.1371/journal.pbio.3003307)
Supplement: S2 Fig — X- and Y-axis represent categories of the taxonomic group and their counts, respectively. The raw data underlying the taxonomic distribution can be found at https://zenodo.org/records/15697913/files/Combined_small_middle_big_datasets.csv (DOCX) [file pbio.3003307.s002.docx]

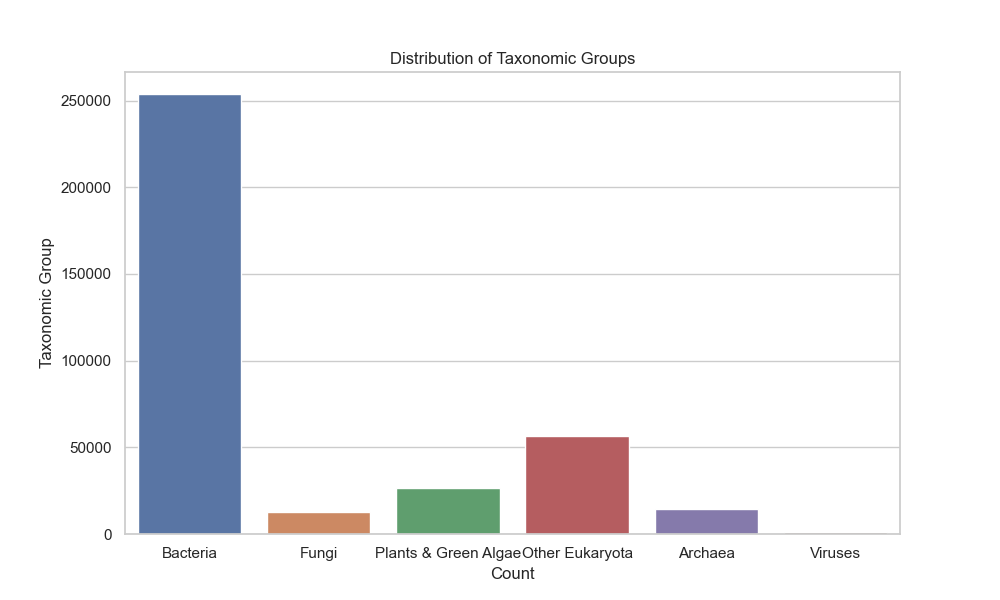


**S2 Fig**: Distribution of taxonomic groups in the reaction-enzyme loose dataset. X- and Y-axis represent categories of the taxonomic group and their counts respectively. The raw data underlying the taxonomic distribution can be found at https://zenodo.org/records/15697913/files/Combined_small_middle_big_datasets.csv
