# Supplementary material for: MEANtools integrates multi-omics data to identify metabolites and predict biosynthetic pathways
Source: PLoS Biol. 2025 Jul 28;23(7):e3003307. doi: 10.1371/journal.pbio.3003307 (PMC12327601; doi:10.1371/journal.pbio.3003307)
Supplement: S4 Fig — The data underlying the distribution of correlation coefficients can be found at https://zenodo.org/records/15697913. (DOCX) [file pbio.3003307.s004.docx]

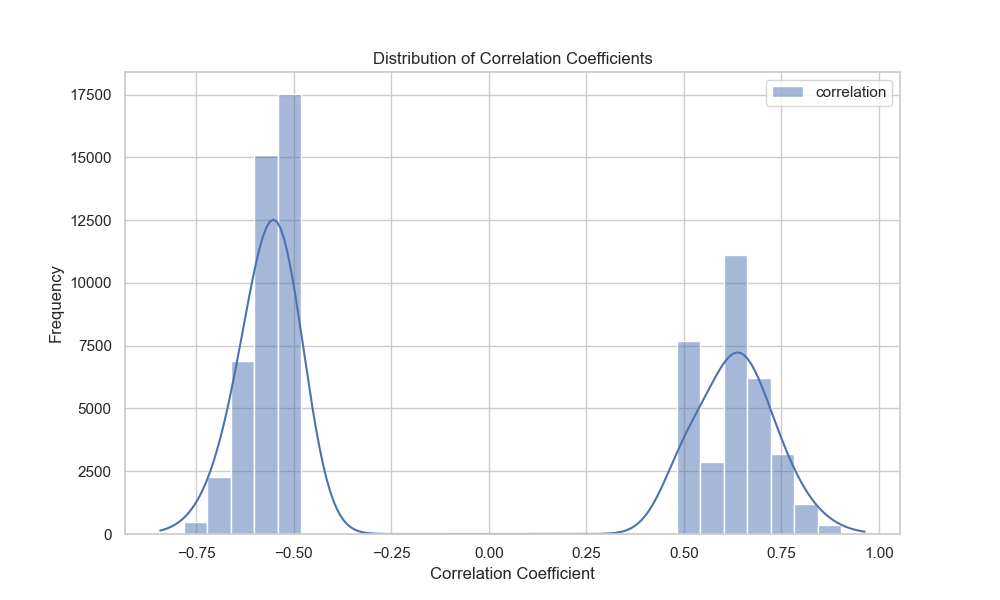


**S4 Fig**: Distribution of correlation coefficients from the correlation analysis between the processed transcriptomics and metabolomics datasets from Jeon *et al*., 2020 [30]. The data underlying the distribution of correlation coefficients can be found at https://zenodo.org/records/15697913.
