# Supplementary material for: MEANtools integrates multi-omics data to identify metabolites and predict biosynthetic pathways
Source: PLoS Biol. 2025 Jul 28;23(7):e3003307. doi: 10.1371/journal.pbio.3003307 (PMC12327601; doi:10.1371/journal.pbio.3003307)
Supplement: S5 Fig — (DOCX) [file pbio.3003307.s005.docx]

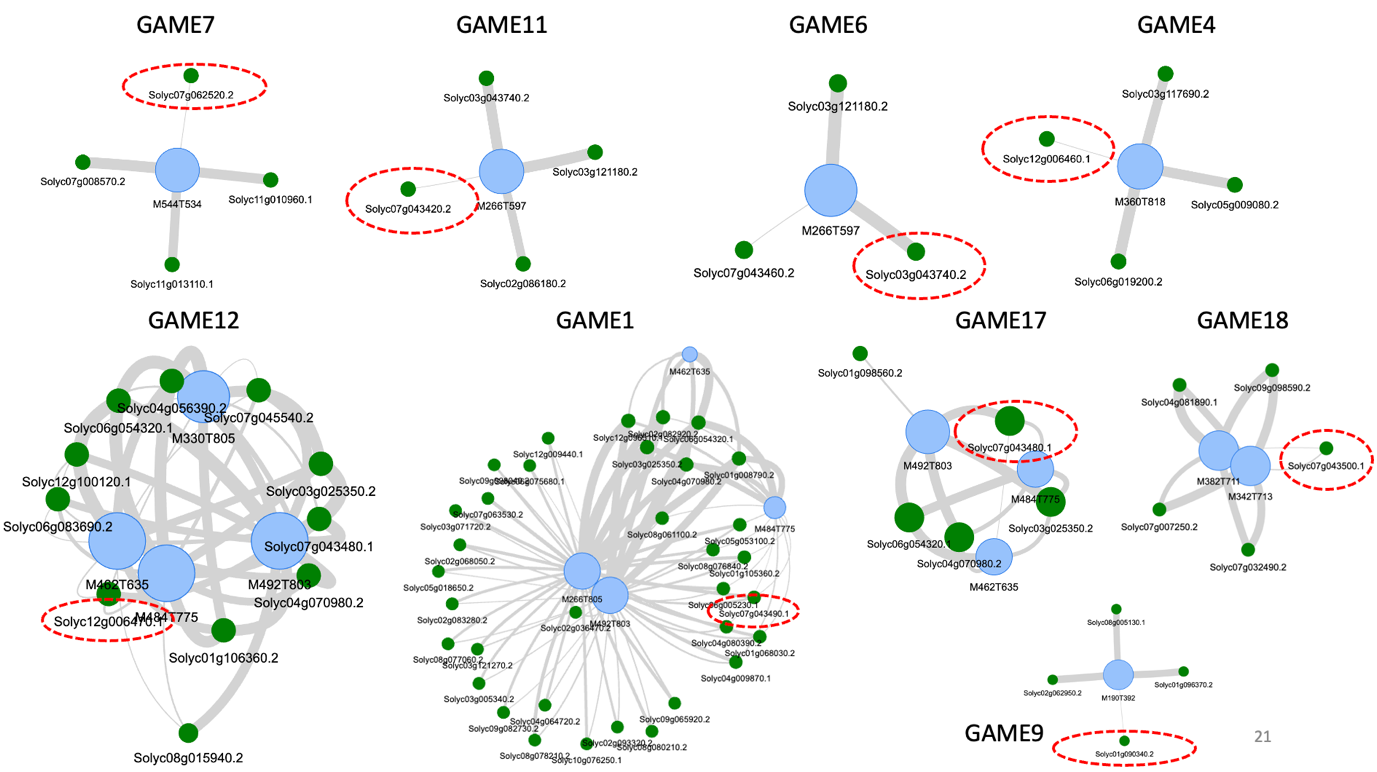


**S5 Fig**: Functional clusters (FC) encompassing genes from the alpha-tomatine pathway of *Solanum lycopersicum*.
