# Supplementary material for: MEANtools integrates multi-omics data to identify metabolites and predict biosynthetic pathways
Source: PLoS Biol. 2025 Jul 28;23(7):e3003307. doi: 10.1371/journal.pbio.3003307 (PMC12327601; doi:10.1371/journal.pbio.3003307)
Supplement: S6 Fig — Number of enzyme associations is reduced while using strict dataset due to its more specific Pfam annotations. Blue arrow shows non-specific enzyme associations predicted with loose dataset. (DOCX) [file pbio.3003307.s006.docx]

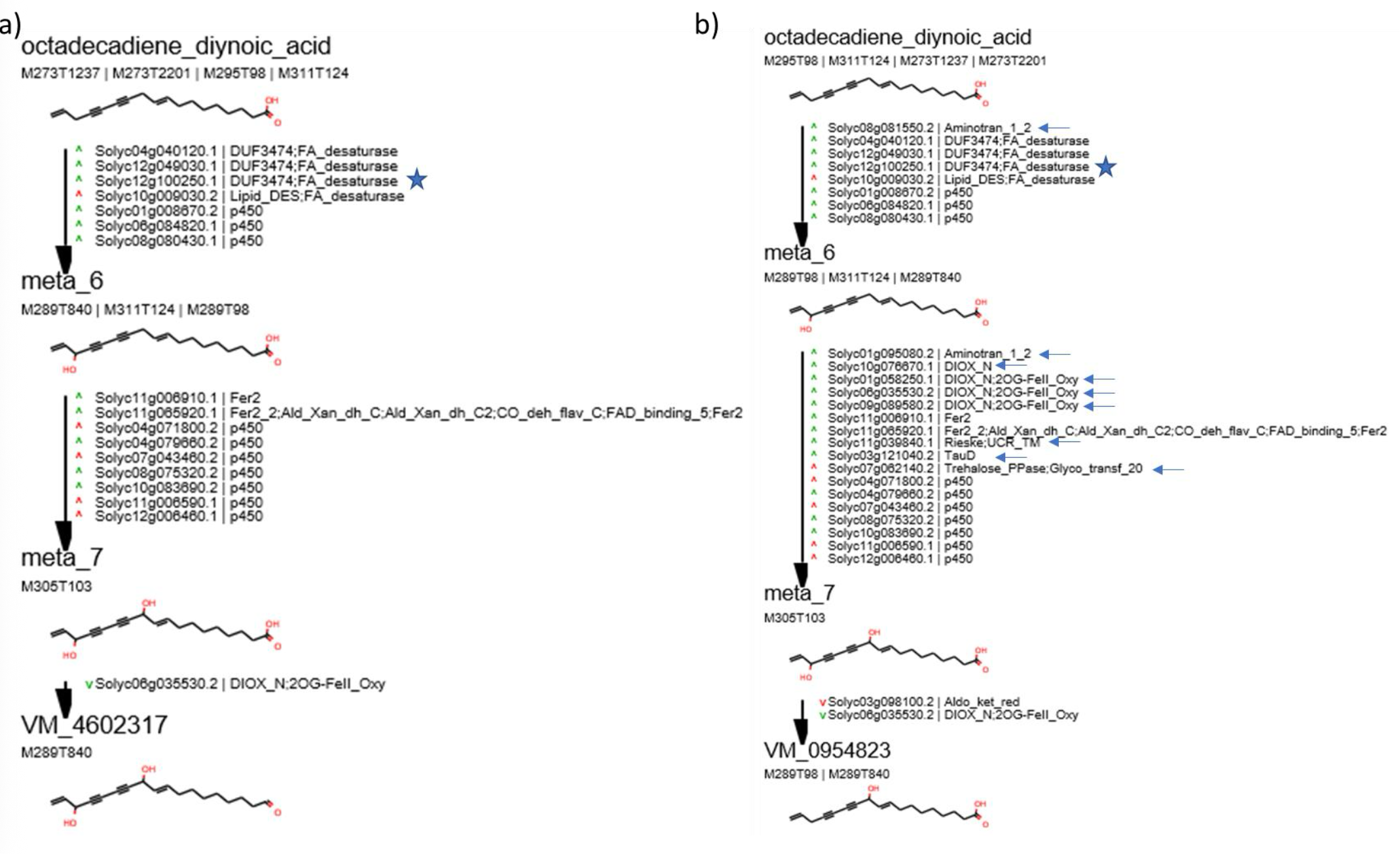


**S6 Fig**: Prediction of intermediate steps of falcarindiol pathway from MEANtools using *strict* (A) and *loose* (B) datasets. Number of enzyme associations is reduced while using *strict* dataset due to its more specific Pfam annotations. Blue arrow shows non-specific enzyme associations predicted with *loose* dataset.
