# Supplementary material for: MEANtools integrates multi-omics data to identify metabolites and predict biosynthetic pathways
Source: PLoS Biol. 2025 Jul 28;23(7):e3003307. doi: 10.1371/journal.pbio.3003307 (PMC12327601; doi:10.1371/journal.pbio.3003307)
Supplement: S7 Fig — The X-axis represents individual decay rates, categorized into treatments involving bacteria, fungi, or a combination of both. The Y-axis denotes the size of the FC, which includes both gene and metabolite features. The results indicate that larger cluster sizes are associated with higher decay rates and larger sample sizes. The data underlying the distribution of FCs size versus decay rates can be found in S5 Data. (DOCX) [file pbio.3003307.s007.docx]

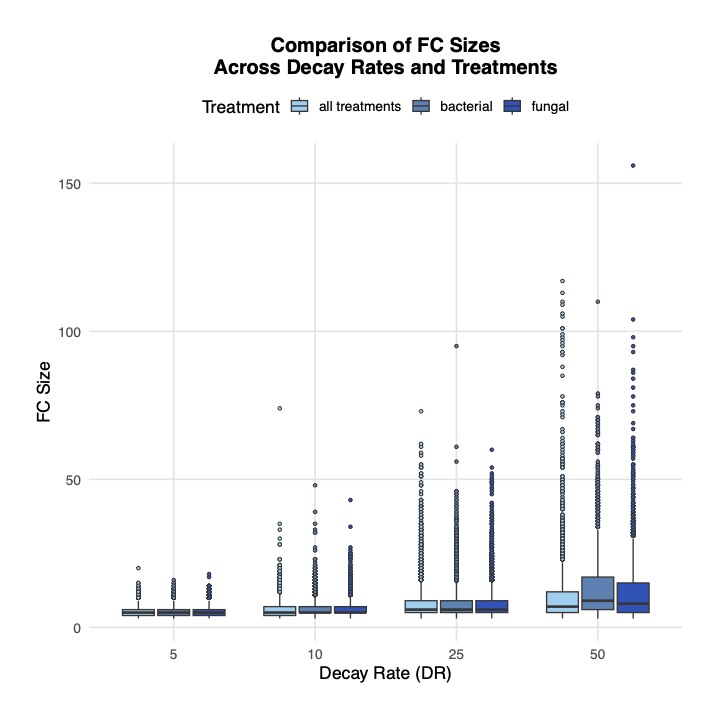


**S7 Fig**: The figure illustrates a comparison of the sizes of various functional clusters (FC) across a range of decay rates and treatments. The X-axis represents individual decay rates, categorized into treatments involving bacteria, fungi, or a combination of both. The Y-axis denotes the size of the FC, which includes both gene and metabolite features. The results indicate that larger cluster sizes are associated with higher decay rates and larger sample sizes. The data underlying the distribution of FCs size vs decay rates can be found in S5 data.
