# Supplementary material for: MEANtools integrates multi-omics data to identify metabolites and predict biosynthetic pathways
Source: PLoS Biol. 2025 Jul 28;23(7):e3003307. doi: 10.1371/journal.pbio.3003307 (PMC12327601; doi:10.1371/journal.pbio.3003307)
Supplement: S8 Fig — Green edges represent coexpression networks and blue edges represent gene-metabolite networks of FCs. Colored nodes represent biosynthetic genes annotated from seven tomato pathways. Coexpression was detected across all treatment dimensions. Coexpression networks and FCs were created with a mutual rank metric and ClusterONE clustering with a decay rate of 10. FCs from the α-tomatine pathway are connected thanks to coexpression edges between the genes GAME12 transaminase (Solyc12g006470), and 2-oxoglutarate-dependent dioxygenase GAME11 (Solyc07g043420), and GAME17 (UDP-glucosyltransferase) (Solyc07g043480) and GAME1 (UDP-galactosyltransferase) (Solyc07g043490). Two genes associated with the biosynthesis of 4-coumarate CoA ligase (4CL) were also connected by coexpression edges in the hydroxy cinnamic acid amide (HCAA) pathway. Merging FCs via coexpression edges between biosynthetic genes was robust across decay rates 10 and 25, with only the connections belonging to the HCAA pathways displayed in decay rate 5. The data underlying this network graph can be found in S6 Data. (DOCX) [file pbio.3003307.s008.docx]

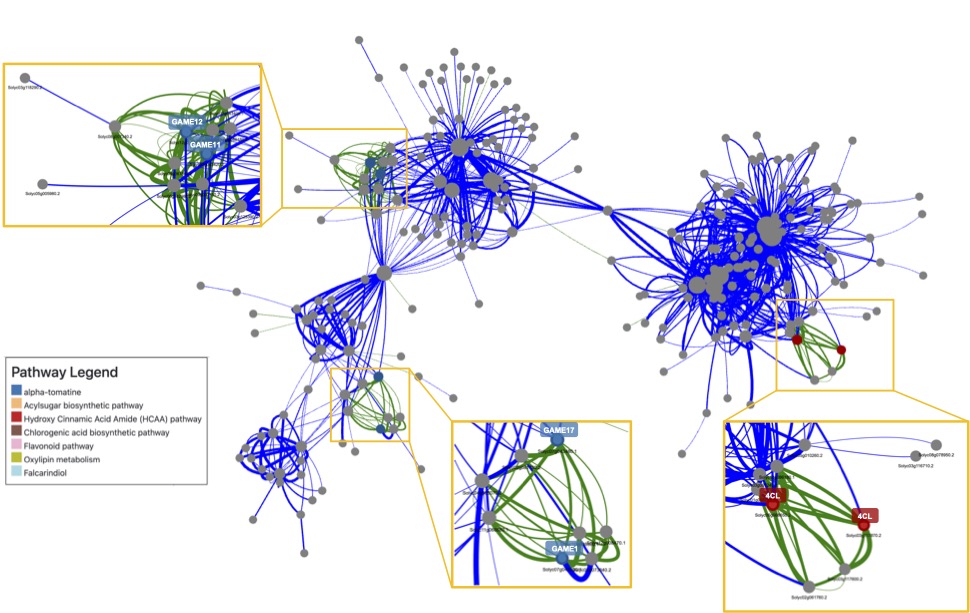


**S8 Fig**: Use of coexpression edges to merge and prioritize FCs. Green edges represent coexpression networks and blue edges represent gene-metabolite networks of FCs. Colored nodes represent biosynthetic genes annotated from seven tomato pathways. Coexpression was detected across all treatment dimensions. Coexpression networks and FCs were created with a mutual rank metric and ClusterONE clustering with a decay rate of 10. FCs from the α-tomatine pathway are connected thanks to coexpression edges between the genes GAME12 transaminase (Solyc12g006470), and 2-oxoglutarate-dependent dioxygenase GAME11 (Solyc07g043420), and GAME17 (UDP-glucosyltransferase) (Solyc07g043480) and GAME1 (UDP-galactosyltransferase) (Solyc07g043490) . Two genes associated with the biosynthesis of 4-coumarate CoA ligase (4CL) were also connected by coexpression edges in the hydroxy cinnamic acid amide (HCAA) pathway. Merging FCs via coexpression edges between biosynthetic genes was robust across decay rates 10 and 25, with only the connections belonging to the HCAA pathways displayed in decay rate 5. The data underlying this network graph can be found in S6 data.
