# Supplementary figures and images for: MEANtools integrates multi-omics data to identify metabolites and predict biosynthetic pathways
Source: PLoS Biol. 2025 Jul 28;23(7):e3003307. doi: 10.1371/journal.pbio.3003307 (PMC12327601; doi:10.1371/journal.pbio.3003307)

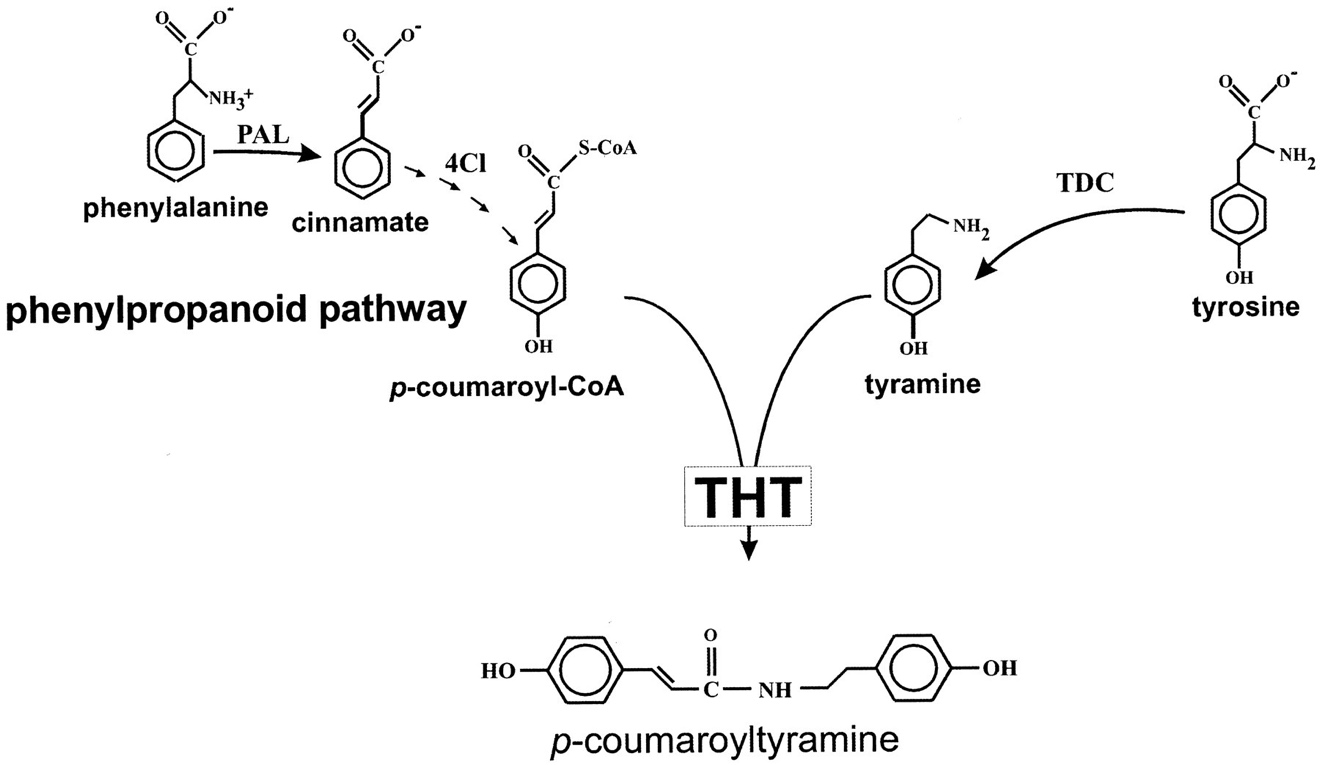


**S9 Fig**: Biosynthetic pathway of *p-coumaroyltyramine* [44].

Supplement: S9 Fig — (DOCX) [file pbio.3003307.s009.docx]
