# Supplementary material for: MEANtools integrates multi-omics data to identify metabolites and predict biosynthetic pathways
Source: PLoS Biol. 2025 Jul 28;23(7):e3003307. doi: 10.1371/journal.pbio.3003307 (PMC12327601; doi:10.1371/journal.pbio.3003307)
Supplement: Attachment — Submitted filename: Response_to_Reviewers.docx [file pbio.3003307.s018.docx]

REVIEWS:

Reviewer #1: The manuscript describes a computational system referred to as MEANtools to predict metabolic pathways based on the reaction rules and metabolite structures stored in public databases. The approach of using transcriptomics and metabolomics data for metabolic pathway prediction would be novel. The validity of this approach is illustrated in a case study involving falcarindiol biosynthesis. However, the use of transcriptomes means that prediction accuracy will decrease if gene annotations are not correct. Currently, annotation curation is still required, so it is questionable whether this method will be widely used.

Response: We appreciate the reviewer’s concern regarding the reliance on gene annotation quality. While it is true that poor annotations can impact prediction accuracy, we would like to emphasize that the same transcriptome data, particularly RNAseq, can be used to refine genome annotation. For example, tools like BRAKER2 (Brůna et al., 2021) use RNAseq data to support or improve gene models in genome assemblies. Furthermore, since MEANtools integrates transcript-metabolite correlations, even incomplete annotations can be augmented by co-expression patterns to highlight candidates involved in metabolic pathways; the algorithm does not depend on fully correct predictions of all intron-exon junctions (even though of course correct predictions are desirable). In addition, metabolite annotation in untargeted metabolomics of plant systems through spectral library matching typically results in a small number of matches (Shen et al., 2019; Shahaf et al., 2016), leaving a substantial portion of the plant chemical space uncharacterized. In this context, integrating transcriptomics helps to prioritize functionally relevant metabolites even when their exact identities remain uncertain. MEANtools leverages transcript-metabolite correlations to reduce this ambiguity, making transcriptomic data especially valuable in cases where metabolite annotation is incomplete or ambiguous. Therefore, we view the use of transcriptome data not as a limitation, but as a valuable resource to both guide and improve annotation, particularly for biosynthetically relevant genes that are the primary focus of our method. We have clarified this point in the revised manuscript in lines 432 - 442.

Other comments:
1. The amount of transcripts does not necessarily correlate with the amount of translation. The authors need to explain why this study uses the transcriptome rather than the proteome.

Response:
We appreciate this important observation and agree that a better explanation would be helpful. In the revised manuscript, we have clarified that transcriptomic data is currently more widely accessible and provides broader coverage than proteomic datasets, particularly in the case of non-model organisms. In one of our published works (Singh et al., 2022), we time-lined the discovery of biosynthetic pathways in plants and observed that transcriptomics has historically played a central role in *de novo* biosynthetic pathway discovery by leveraging co-expression patterns of pathway genes. While we recognize that transcript levels do not perfectly correlate with protein abundance, transcriptomics often serves as a sufficiently reliable proxy for gene activity across many biological contexts. Additionally, the modular nature of MEANtools allows for the integration of proteomic data, provided that protein annotations and their abundance profiles are available across samples. In this study, we focused on transcriptomic data, mainly due to its current availability, which is why proteomics was not emphasized in the manuscript. Nonetheless, we have modified the text in the manuscript (in lines 438 - 442) by highlighting the possibility of using proteomics data. Once more (plant) proteomics data will become available, the addition of proteomics modules to MEANtools will be an interesting next step.

2. The authors mentioned that MEANtools can assign potential structure matches by identifying adducts in the metabolome and querying the LOTUS database for matching metabolites based on molecular weight. It would be better if there was a detailed explanation of what kind of compound data is being used. For example, what exactly is an adduct? There are various types of adducts of ions detected, and multiply charged ions should also be detected.

Response:
We thank the reviewer for this valuable suggestion. In the revised manuscript, we have included a more detailed explanation of adduct formation and its relevance in MEANtools in lines 529 - 551. Briefly, adducts are complexes formed between a molecule and an ion or neutral species during ionization in mass spectrometry, causing the observed m/z value to differ from the neutral molecular mass. To account for this, MEANtools matches experimental masses against known compounds while considering common adduct types. Since adduct formation varies depending on the type of mass spectrometer used, MEANtools allows users to specify adducts relevant to their platform. If such information is not provided, MEANtools uses a default list of approximately 40 commonly observed adducts, curated from diverse MS instruments. MEANtools currently treats all mass features as neutral and does not support multiply charged ions. We appreciate this suggestion and plan to enhance the codebase in the next release to accommodate features for handling multiple charge states.

3. When estimating the structures of metabolic compounds, it may also be necessary to consider the analytical accuracy required of the mass spectrometer. Will data from a time-of-flight mass spectrometer with high mass accuracy be required?

Response:

We appreciate the reviewer’s observation regarding the influence of instrument resolution on structural inference. In the revised manuscript (line 540 - 551), we now clarify that MEANtools benefits from high-resolution MS1 data, such as that generated by time-of-flight (TOF) or Orbitrap platforms. Accurate adduct matching and molecular formula estimation benefit significantly from such resolution. While high-resolution instruments enable more precise structural mapping, lower-resolution data can increase the likelihood of false positives. To accommodate different instrumentation, MEANtools includes a user-adjustable ppm (parts per million) tolerance parameter, with a default of 20 ppm. Users working with ultra-high-resolution data can reduce this threshold (e.g., to 5 ppm) to further enhance matching specificity. This clarification has been added to the revised Methods section.

4. On line 268, the authors mention that MEANtools also anticipated steps five and six of the pathways. Has a system capable of predicting metabolic pathways that include many steps like this been reported? If not, the authors may wish to emphasize this point as an advantage of their research.

Response:

This is an excellent suggestion. Although this has been covered in detail in the Methods section under “Easy-to-browse MEANtools output”, we have now highlighted MEANtools’ ability to infer multi-step biosynthetic routes as a unique feature and discuss this approach in the Results section (in lines 302 - 313). This ability stems from MEANtools’ integration of recursive reaction rules, correlation-driven filtering, which collectively enable longer biosynthetic paths to be proposed and refined and the use of directed acyclic graphs (DAGs) that identifies reaction paths in the network.

5. PAL, 4CL, THT are abbreviations. Therefore the full name should be used the first time it appears in the text body. In addition, it should be better to show the metabolic pathway including PAL, 4CL and THT.

Response:

We thank the reviewer for spotting this issue. We have added the full name of all enzymes along with the metabolic pathway that involves PAL, 4CL and THT as a Supplementary image (S9 Fig).


Reviewer #2: The authors developed MEANtools, a computational tool designed for metabolomics and systems biology studies. This tool integrates transcriptomics (gene expression data) and metabolomics (mass spectrometry data) to identify and predict metabolic pathways, providing a powerful framework for exploring biosynthetic processes.

In systems biology research, the integration of omics data is crucial for understanding complex metabolic interactions, yet this manuscript presents an important contribution by offering clear guidance on how transcriptomic and metabolomic datasets can be effectively combined.
While the manuscript is well-written, it could benefit from further clarity in explaining how the the relationship between transcripts and mass signatures modifies or refines existing biosynthetic hypotheses, particularly as illustrated in Figure 4.

Response:
We thank the reviewer for this insightful comment. In the revised manuscript, we have elaborated on how MEANtools leverages transcript-metabolite correlations to refine biosynthetic hypotheses (in lines 498 - 513). Specifically, Figure 4 illustrates the co-expression and -abundance patterns of genes and metabolites within the PAL and THT biosynthetic pathways. The strength of MEANtools lies in its use of mutual rank statistics, which enhances the interpretation of correlation values by incorporating expression rank consistency across samples. A key example is the association between the gene Solyc08g068790.1 and the metabolite M316T772. While this pair shows a strong Pearson correlation coefficient of 0.87, it is the mutual rank score of 0.39 that further refines this association by highlighting variable co-expression patterns. Notably, although all four genes in the THT module exhibit high correlation with M316T772, their expression profiles vary significantly across time points. This subtle but important distinction is effectively captured by mutual rank scoring, allowing MEANtools to better prioritize transcript-metabolite pairs likely involved in the same biosynthetic pathway. We have now explicitly explained this mechanism in the Results section to improve clarity.

The use of mass shift to predict chemical reactions has also been used by Jourdan et al in 2008 (doi:10.1093/bioinformatics/btm536), could be interesting to add this reference.

Response:

We thank the reviewer for highlighting this relevant work. In the revised manuscript, we now cite both the MetaNetter-related publication, Jourdan et al. (2008) and introduced MetaNetter as a tool that introduced the use of mass-shifts by incorporating known biochemical transformations for generating reaction-based metabolic networks. This addition has been made in the Introduction (lines 73–77) to better contextualize MEANtools’ novelty, which lies in its integration of multi-omics data and its combined use of mass-shift analysis with reaction rule-based pathway prediction.

The presented code works well but its integration into one of the multiple online tools developed by the authors could help future users.

Response:

We appreciate the reviewer’s positive feedback on the code usability. In the future, we do plan to work towards a web-based interface that will allow users to upload their transcriptomic and metabolomic data for automated analysis. As a part thereof, we will perform a feasibility check related to managing multi-omics data on a local server. If this turns out to be feasible, a web-version of the MEANtools will be one of the next steps.

Based on these comments, I would recommend the acceptance of this work.
